# Equilibrio ocupacional, discapacidad y funcionalidad en personas con enfermedades reumáticas

**DOI:** 10.23938/ASSN.1053

**Published:** 2023-12-28

**Authors:** Ana M Martín Pérez, Nuria Máximo-Bocanegra, Francisco Rivas Ruiz, Isabel M Alguacil-Diego, Rosa M Martínez-Piédrola

**Affiliations:** 1 Universidad Rey Juan Carlos Universidad Rey Juan Carlos Departamento de Fisioterapia, Terapia Ocupacional, Rehabilitación y Medicina física Madrid Spain; 2 Hospital Costa del Sol Unidad de Investigación e Innovación Marbella Málaga Spain; 3 RICAPPS-Red de Investigación en Cronicidad, Atención Primaria y Prevención y Promoción de la Salud Spain

**Keywords:** Equilibrio Ocupacional, Discapacidad, Funcionalidad, Enfermedad Reumática, Occupational Balance, Disability, Functionality, Rheumatic Disease

## Abstract

**Fundamento:**

El objetivo del presente estudio es conocer el equilibrio ocupacional de las personas con enfermedad reumática y analizar su relación con la participación, el rendimiento y satisfacción con las actividades de la vida diaria (AVD), así como evaluar si la edad o recibir tratamiento no farmacológico influyen en los resultados.

**Método:**

Estudio transversal realizado entre marzo y noviembre de 2021 en personas con diagnóstico de enfermedad reumática en fase no avanzada procedentes de la Asociación *ConArtritis*, seleccionadas mediante muestreo aleatorio simple. Se recogieron, *on-line* y/o por teléfono, datos sociodemográficos y puntuaciones de los cuestionarios OBQ, IMPACT-S, COPM, y de un cuestionario creado ad hoc para las AVD.

**Resultados:**

Los 47 participantes no presentaban un buen equilibrio ocupacional (OBQ: 34,2; DE: 13,7). A pesar de una buena participación en AVD (IMPACT-S: 76,8; DE: 13,1), el grado de desempeño y de satisfacción con las AVD distó de ser óptimo (COPM-R: 3,9; DE=2,0 y COPM-S: 4,3; DE=2,5). El 46,8% encontraba limitaciones en al menos cuatro AVD, tanto básicas como instrumentales, y el 61,7% utilizaba al menos un producto de apoyo en su día a día. Estas limitaciones disminuían su tiempo de descanso y afectaban a su actividad laboral, aficiones y relaciones personales. El grado de desempeño se relacionó negativamente con la edad (p=0,04); recibir tratamiento no farmacológico no modificó las puntuaciones.

**Conclusión:**

Los datos recabados sugieren que las personas con enfermedad reumática en fases no avanzadas de la enfermedad perciben que su equilibrio ocupacional mejoraría si encontrasen menos limitaciones en las AVD.

## Abstract (en)

**Background:**

We aimed to assess the occupational balance of people with rheumatic disease, analyze its relationship with participation, performance, and satisfaction with daily life activities, and evaluate whether age or receiving non-pharmacological treatment affects the outcome.

**Methods:**

Cross-sectional study carried out between March and November 2021; patients with non-advanced stage of rheumatic disease from the *ConArtritis* Association -selected through simple random sampling-, were included. Sociodemographic data and scores from the OBQ, IMPACT-S, COPM questionnaires, and a questionnaire created *ad hoc* for daily life activities were collected online and/or by telephone.

**Results:**

The occupational balance of the 47 participants was low (OBQ: 34.2; SD: 13.7). Despite a high participation in daily life activities (IMPACT-S: 76.8; SD: 13.1), the degree of performance and satisfaction with these activities was far from optimal (COPM-R: 3.9; SD: 2.0 and COPM-S: 4.3; SD: 2.5); 46.8% of the participants found limitations in at least four daily life activities (basic and instrumental) and 61.7% used at least one support product in their daily lives. These limitations reduced their resting time and affected their jobs, hobbies, and personal relationships. The level of performance was negatively related to age (p=0.04); No changes in the scores were found in patients who received non-pharmacological treatment.

**Conclusion:**

Our results suggest that individuals with non- advanced stage of a rheumatic disease perceive that their occupational balance may be improved should they have fewer limitations in daily life activities.

## INTRODUCCIÓN

Las enfermedades reumáticas abarcan un amplio espectro de trastornos del tejido articular, musculoesquelético y conectivo, habiéndose descrito más de 200 hasta la fecha^1^. Pueden causar incapacidad funcional, generando un gran impacto a nivel económico, social y personal^2^.

De acuerdo con la Organización Mundial de la Salud, el 20% de la población mundial tiene algún tipo de enfermedad reumática, que es el segundo motivo de ausentismo laboral y representa el 35% de las causas de invalidez total o parcial de la población adulta del mundo occidental^3^. La incidencia global es de dos mujeres por cada hombre^4^. En la Unión Europea afectan a más de 120 millones de personas de todas las edades^4^, mientras que en España se estima que las padecen el 22,6% de la población y que suponen el 10-15% de las consultas de atención primaria y el 10% de las hospitalarias, siendo la causa del 19% de las incapacidades laborales^4^.

Las enfermedades reumáticas limitan en gran medida la destreza y la movilidad, impulsan a jubilaciones anticipadas, reducen la riqueza acumulada e impactan en la capacidad de participación de la vida social^3^. Cuando afectan a los miembros superiores, presentan un desafío particularmente difícil para el desempeño de la vida cotidiana^5^. De hecho, el uso funcional de las manos es considerado un componente valioso de la identidad humana, ya que son el interfaz de una persona con el mundo que le rodea^5^. La existencia de lesiones graves en la mano, de malestar crónico y de dolor se correlacionan con la disminución de la calidad de vida, la depresión y la ansiedad^5^. Por ello, se considera necesario crear programas de participación en la comunidad, de educación de profesionales y pacientes y de detección precoz de la enfermedad así como ofrecer tratamientos rehabilitadores eficaces^6^.

En el campo de la Terapia Ocupacional (TO) existen dos conceptos que podrían ayudar a diseñar mejores programas de rehabilitación en esta área: el perfil y el equilibrio ocupacional.

El perfil ocupacional permite conocer la historia y experiencias ocupacionales de la persona: patrones de vida diaria, valores, intereses, necesidades, preocupaciones y problemas en relación a la realización de las ocupaciones y actividades de la vida diaria (AVD) y sus prioridades^7^. Esta información facilita establecer un programa de TO acorde a sus intereses y necesidades que mejore la participación y, por lo tanto, su salud en términos globales.

El equilibrio ocupacional se refiere al uso del tiempo en diferentes actividades categorizadas en autocuidado, descanso, educación, trabajo, juego, ocio y tiempo libre y participación social. Es biológicamente necesario para mantener el ritmo de vida interno y externo y alcanzar un buen estado de salud^8^. La persona con enfermedad reumática habitualmente encuentra barreras para alcanzar el equilibrio ocupacional, realizando muchas de las actividades deseadas con sobreesfuerzo o claudicando en el intento^9^.

Una revisión sistemática sobre diversas intervenciones de TO en artritis reumatoide señala que los terapeutas ocupacionales tienen un rol clave en la intervención, y que esta es efectiva, con resultados positivos en la reducción del dolor y en la mejora de la funcionalidad y la calidad de vida^10^. Es necesario, pues, ahondar en aquellas intervenciones basadas en la evidencia científica que pudieran repercutir favorablemente en el perfil y el equilibrio ocupacional, para lo que es necesario conocer previamente el impacto de estas enfermedades en el equilibrio ocupacional; en nuestro conocimiento, no se han realizado estudios sobre el equilibrio ocupacional y la enfermedad reumática en España.

Por ello, el objetivo principal del presente estudio es conocer y analizar el equilibrio ocupacional de las personas con enfermedad reumática e investigar su relación con la participación, el rendimiento y la satisfacción con las AVD. El objetivo secundario ha sido evaluar si la edad o recibir tratamiento no farmacológico influye en los resultados de las evaluaciones empleadas.

## MATERIAL Y MÉTODOS

Se trata de un estudio observacional transversal realizado en personas con diagnóstico de enfermedad reumática.

El reclutamiento de pacientes comenzó en marzo de 2021, mediante muestreo aleatorio simple de personas procedentes de la Asociación Coordinadora Nacional de Artritis, *ConArtritis*. Se incluyeron personas de ambos sexos, mayores de 18 años, que hubieran firmado el consentimiento informado para su inclusión en la investigación; se excluyeron aquellas con diagnóstico de enfermedad reumática en estadio avanzado (no poder realizar ninguna AVD de manera independiente) o con deterioro de la capacidad cognitiva (*Mini Mental State Examination* < 24 puntos).

Mediante correo electrónico se recogieron datos sociodemográficos (edad y sexo) y clínicos (zona del cuerpo más afectada por la enfermedad y estar recibiendo en el momento actual tratamiento no farmacológico).

Las medidas de resultado fueron recogidas por el mismo evaluador telemáticamente y mediante entrevistas telefónicas.

Las medidas de valoración empleadas fueron:

- *Occupational Balance Questionnaire* (OBQ): Se utilizó la versión española adaptada y validada. Evalúa la satisfacción de la persona con la variedad y cantidad de tareas, ocupaciones, y actividades que realiza en su vida cotidiana y el tiempo que dedica a cada una de ellas^11^. Cuenta con 13 afirmaciones y seis calificaciones posibles, desde 0=completamente en desacuerdo, hasta 5=completamente de acuerdo. La puntuación máxima es 65 puntos: a mayor puntuación, mayor equilibrio ocupacional.
- *ICF-Measure of Participation and Activities Screener* (IMPACT-S): Autoinforme basado en la Clasificación de Funcionamiento, Discapacidad y Salud (CIF) que evalúa las limitaciones percibidas en las actividades y la participación. Consta de nueve dominios, uno por cada capítulo de la CIF, sobre actividades y participación con 32 elementos cuyo rango de opciones de respuesta varía entre 0=no se puede realizar la actividad y 3=sin limitación. Las puntuaciones se convierten a una escala de 0 a 100, donde una mayor puntuación indica mayor nivel de participación. El IMPACT-S muestra buena reproducibilidad, moderada sensibilidad y buena fiabilidad test-retest y consistencia interna^12^.
- Medida Canadiense del Desempeño Ocupacional o COPM, del inglés *Canadian Occupational Performance Measure*: Es una medida de resultados centrada en el cliente para que las personas identifiquen y prioricen los problemas cotidianos que restringen su participación en la vida cotidiana. Esta medida se centra en el desempeño ocupacional en todos los ámbitos de la vida, incluido el autocuidado, el ocio y la productividad. La persona prioriza los problemas según una escala visual que varía de 1=nada importante a 10=muy importante. Una vez seleccionados los cinco problemas más importantes, valora del 1 al 10 cómo es su ejecución o desempeño y el grado de satisfacción que le produce esa situación, obteniendo dos índices finales: desempeño (COPM-R) y satisfacción (COPM-S)^13^. Las puntuaciones totales se obtienen dividiendo la suma de los valores de desempeño o satisfacción entre el número de problemas; A mayor puntuación, mayor será el grado de desempeño y de satisfacción. Ganancias de más de 2 puntos son clínicamente importantes^13^.
- Cuestionario tipo Likert, autoadministrado, creado *ad-hoc* para conocer la autopercepción de las personas participantes sobre las limitaciones y la capacidad en diferentes áreas de ocupación (AVD, descanso, trabajo, ocio y participación social). Diseñado atendiendo a las recomendaciones para el diseño y validación de cuestionarios en Ciencias de la Salud^14^, no ofrece una puntuación global y consta de nueve preguntas, tres dicotómicas y seis de opción múltiple.

El estudio fue aprobado por el Comité de Ética de la Investigación de la Universidad Rey Juan Carlos (número de registro interno: 2001202104021) de acuerdo con los principios éticos para las investigaciones médicas en seres humanos de la Declaración de Helsinki y posteriores revisiones^15^.

Las variables cuantitativas se describieron con media y desviación estándar (DE), y las cualitativas con frecuencia absoluta y porcentaje. Para correlacionar dos variables cuantitativas, se utilizó el coeficiente de correlación de Pearson. La normalidad de la distribución de datos se comprobó mediante el test de Kolmogórov-Smirnov; y se utilizaron los test U de Mann-Whitney y Kruskal Wallis para valorar diferencias en variables de resultado cuantitativas entre dos o más grupos. El nivel de significación estadística se estableció en p<0,05. El análisis estadístico se llevó a cabo con el programa estadístico IBM SPSS Statistics versión 28.

## RESULTADOS

Aceptaron participar en el estudio 50 sujetos. Tres no cumplían con los criterios de inclusión, por lo que la muestra quedó conformada finalmente por 47 participantes. De ellos, 43 eran mujeres (91,5%). La edad media de la muestra fue de 47,9 años (DE: 10,9; rango: 30-68).

La tabla 1 recoge las características clínicas de la muestra. Algo más de la mitad de pacientes (55,3%) presentan artritis reumatoide, siendo las manos la región más afectada (87,2%). Más de la mitad de pacientes recibían tratamiento no farmacológico (55,3%) y, de ellos, cerca de la mitad recibían fisioterapia (42,9%). Las tres personas con espondiloartritis fueron más jóvenes y todas recibían tratamiento no farmacológico.

**Tabla 1:** Características clínicas de la muestra

	Enfermedad reumática				
Variables	AR	AIJ	APs	EA	Global
	n=26 (55,3%)	n=10 (21,3%)	n=8 (17%)	n=3 (6,4%)	n=47
	n (%)	n (%)	n (%)	n (%)	n (%)
Sexo (mujer)	24 (92,3)	10 (100)	6 (75,0)	3 (100)	43 (91,5)
Edad*	51,8 (10,7)	45,7 (9,1)	44,5 (10,1)	33,0 (3,0)	48,0 (10,9)
Parte del cuerpo más afectada					
Manos	24 (92,3)	9 (90,0)	7 (87,5)	1 (33,3)	41 (87,2)
Codos	6 (23.1)	5 (50,0)	4 (50,0)	0 (0,0)	15 (31,9)
Hombros	7 (26,9)	7 (70,0)	5 (62,5)	0 (0,0)	19 (40,5)
Tobillos	15 (57,7)	7 (70,0)	7 (87,5)	0 (0,0)	29 (61,7)
Rodillas	11 (42,3)	7 (70,0)	4 (50,0)	0 (0,0)	22 (46,8)
Cadera	5 (19,3)	5 (50,0)	5 (62,5)	1 (33,3)	16 (34,0)
Mandíbula	1 (3,8)	0 (0,0)	0 (0,0)	0 (0,0)	1 (2,1)
Columna	2 (7,7)	4 (40,0)	5 (62,5)	1 (33,3)	12 (25,6)
Tratamientos no farmacológicos					
No reciben	14 (53,8)	6 (60,0)	1 (12,5)	0 (0,0)	21 (44,7)
Fisioterapia	3 (11,5)	2 (20,0)	2 (25,0)	2 (66,7)	9 (19,1)
Psicología	3 (11,5)	2 (20,0)	1 (12,5)	0 (0,0)	6 (12,8)
Podología	2 (7,7)	0 (0,0)	0 (0,0)	0 (0,0)	2 (4,3)
Reciben >1	4 (15,4)	0 (0,0)	4 (50,0)	1 (33,3)	9 (19,1)

*: media (desviación estándar); AR: artritis reumatoide; AIJ: artritis idiopática juvenil; APs: artritis psoriásica; EA: espondiloartritis.

Las puntuaciones obtenidas en el OBQ indicaron que los participantes no presentaban un buen equilibrio ocupacional (34,2 sobre 65). A pesar de que la puntuación media obtenida en la escala IMPACT-S (76,8 sobre 100) reflejó que la participación en actividades fue equilibrada, las puntuaciones medias de las escalas COPM-R y COPM-S (3,9 y 4,3 sobre 10, respectivamente) señalaron que ni el grado de desempeño ni el grado de satisfacción con los resultados de las actividades que realizan diariamente eran óptimos (Tabla 2). El grado de participación en actividades varió significativamente entre tipos de enfermedad: las personas con artritis psoriásica y con artritis reumatoide obtuvieron puntuaciones en la escala IMPACT-S más altas que el resto.

**Tabla 2:** Puntuaciones en las medidas de resultado según el tipo de enfermedad reumática

Cuestionario	Puntuación					p*
	Media (DE)					
	AR	AIJ	APs	EA	Global	
	(n=26)	(n=10)	(n=8)	(n=3)	(n=47)	
OBQ	37,5 (12, 7)	30,4 (16,8)	29,0 (12,8)	32,3 (15,7)	34,2 (13,7)	0,643
IMPACT-S	80,0 (11,9)	69,7 (12,3)	69,3 (13,9)	90,3 (2,1)	76,8 (13,1)	0,016
COPM-R	4,0 (2,2)	4,0 (2,4)	3,7 (1,4)	4,1 (1,2)	3,9 (2,0)	0,988
COPM-S	4,4 (2,6)	4,8 (2,7)	3,5 (1,8)	3,8 (3,2)	4,3 (2,5)	0,431

DE: desviación estándar; AR: artritis reumatoide; AIJ: artritis idiopática juvenil; AS: artritis psoriásica; EA: espondiloartritis; *: Kruskal-Wallis; OBQ: *Occupational Balance Questionnaire*; IMPACT-S: *ICF Measure of Participation and Activities questionnaire*; COPM-R: Medida Canadiense del Desempeño Ocupacional - Desempeño; COPM-S: Medida Canadiense del Desempeño Ocupacional - Satisfacción.

**Tabla 3:** Puntuaciones en las medidas de resultado en relación con el tratamiento no farmacológico

Cuestionario	Puntuación		p*
	Media (DE)		
	Tratamiento no farmacológico		
	No	Sí	
	n=21 (44,7%)	n=26 (55,3%)	
OBQ	34,4 (14,8)	34,0 (13,0)	0,458
IMPACT-S	76,6 (14,2)	77,0 (12,5)	0,461
COPM-R	3,9 (2,3)	4,1 (1,7)	0,368
COPM-S	4,7 (2,6)	4,0 (2,4)	0,159

DE: desviación estándar; *: U de Mann Whitney; OBQ: *Occupational Balance Questionnaire*; IMPACT-S: *ICF Measure of Participation and Activities questionnaire*; COPM-R: Medida Canadiense del Desempeño Ocupacional - Desempeño; COPM-S: Medida Canadiense del Desempeño Ocupacional - Satisfacción.

El análisis estadístico no mostró que las puntuaciones obtenidas en los diferentes cuestionarios se relacionaran con realizar terapias no farmacológicas (Tabla 3).

A mayor edad, la puntuación en el COPM-R fue menor, indicando un menor grado de realización (r=-0,410; p=0,04); la edad no se relacionó con ninguna otra medida.

El análisis también indica las puntuaciones obtenidas en las distintas medidas se correlacionaron significativamente: a mayor percepción de ausencia de limitaciones (mayores puntuaciones en el IMPACT-S), mayor desempeño en las actividades diarias (mayores valores en el COPM-R), mayor satisfacción por dicho desempeño (valores más altos en el COPM-S) y mayor equilibrio ocupacional (mayores puntuaciones en OBQ) (Tabla 4).

**Tabla 4:** Correlaciones entre las puntuaciones obtenidas en los distintos cuestionarios del estudio

	IMPACT-S	COPM-R	COPM-S
OBQ-E	r=0,502	r=0,335	r=0,478
	p<0,001	p=0,021	p=0,001
IMPACT-S		r=0,497	r=0,396
		p<0,001	p=0,006
COPM-R			r=0,679
			p<0,001

r: coeficiente de correlación de Pearson; COPM-R: Medida Canadiense del Desempeño Ocupacional - Desempeño; COPM-S: Medida Canadiense del Desempeño Ocupacional - Satisfacción; IMPACT-S: ICF *Measure of Participation and Activities questionnaire*; OBQ-E:Versión española del *Occupational Balance Questionnaire*.

La Tabla 5 recoge los resultados del cuestionario tipo Likert desarrollado por los autores para valorar la autopercepción en las posibles limitaciones que presentan en diferentes actividades cotidianas. La muestra percibe su equilibrio ocupacional como adecuado pero mejorable si encontrasen menos limitaciones en algunas AVD como hacer la cama, recoger la mesa o fregar los platos, entre otras. Además, describen que estas limitaciones les impiden tener un sueño adecuado, presentando alteraciones del sueño que disminuyen el tiempo de descanso. Igualmente, perciben afectación en la ejecución de su actividad laboral o en sus relaciones personales. El 80,9% declara no dedicar el tiempo suficiente a sus aficiones. Casi la mitad de los participantes (46,8%) encuentran limitaciones en al menos cuatro AVD, tanto básicas (vestirse, comer, levantarse o acostarse de la cama) como instrumentales (comprar, limpiar, cocinar o conducir) y más de la mitad (61,7%) utiliza al menos un producto de apoyo en su día a día (abridores de tarros, alza de inodoro o bastones) y el 10,6% más de 4. Un 95,8% (n=45) declaró que las actividades en las que presentan limitaciones tienen una importancia alta para ellos. La mayoría (66%) reconocen poder realizar las actividades cotidianas a pesar de presentar dificultades debido a las restricciones de movilidad de una o más articulaciones.

**Tabla 5:** Autopercepción de las limitaciones en actividades cotidianas.

Área de ocupación	n (%)
Actividades básicas de la vida diaria: limitaciones	
Sin limitación	11 (21,3)
En ≤ 3 actividades	14 (29,8)
En ≥ 4 actividades	22 (46,8)
Actividades instrumentales de la vida diaria: limitaciones	
Sin limitación	7 (14,9)
En ≤ 3 actividades	18 (38,3)
En ≥ 4 actividades	22 (46,8)
Productos de apoyo: utilización	
Ninguno	18 (38,3)
≤ 3	24 (51,1)
≥ 4	5 (10,6)
Sueño y descanso: afectación	
Sí	39 (83)
No	8 (17)
Actividad laboral: afectación	
Sí	37 (78,7)
No	10 (21,3)
Ocio: dedicación del tiempo deseados	
Sí	9 (19,1)
No	38 (80,9)
Relación familiares y amistades: afectación	
Ninguna	24 (51,1)
Ha mejorado	4 (8,5)
Ha empeorado	19 (40,4)
Actividades con limitaciones: importancia	
Mucha	20 (42,6)
Algo	25 (53,2)
Ninguna	2 (4,3)
Frase que más se adecua a su situación actual:	
Tengo capacidad para realizar...	
... todas las actividades cotidianas sin presentar dificultad	3 (6,4)
... todas las actividades cotidianas a pesar de presentar dificultades debido a las restricciones de movilidad de una o más articulaciones	31 (66)
... únicamente algunas actividades cotidianas, o tengo incapacidad total	13 (27,7)

## DISCUSIÓN

El equilibrio ocupacional de las personas se relaciona con la cantidad de ocupaciones y la variedad de estas; es un concepto subjetivo porque la propia percepción de la persona determina si posee o no ese equilibrio. Según Wagman y col^16^, el equilibrio ocupacional se puede describir en términos de compensación de la cantidad de actividades destinadas a las distintas áreas ocupacionales, en relación con la variabilidad de las ocupaciones de diferentes características y en relación con el uso del tiempo destinado a esas actividades.

Atendiendo a este tipo de conceptualización vemos cómo nuestros datos constatan que la muestra, según el cuestionario IMPACT-S, posee un buen equilibrio ocupacional pero bajas puntuaciones en la satisfacción y el desempeño valorado con el COMP. Así pues, su percepción respecto a su equilibrio ocupacional es negativa. Esto resulta fundamentalmente para un desempeño ocupacional cuya realización y satisfacción era baja. Este desempeño es percibido como inadecuado en las AVD, principalmente por dos razones, bien por la necesidad de utilizar productos de apoyo o bien requerir más tiempo para su realización.

Esa percepción de la necesidad de tomar más tiempo en la realización de las actividades influye en el equilibrio ocupacional, ya que el tiempo dedicado a ciertas tareas va en detrimento de otras. Si, además, la satisfacción no es buena, el impacto negativo en la calidad de vida será un hecho, ya que a mayor equilibrio ocupacional, mayor será la calidad de vida^10^. Al igual que en nuestro estudio, otros autores observaron cómo la enfermedad reumática afecta menos a las AVD básicas que al ocio o a las actividades sociales^17^^,^^18^, con una mayor repercusión en las actividades instrumentales y, por lo tanto, un desequilibrio ocupacional. Otro aspecto importante a valorar con repercusión en el equilibrio ocupacional tiene que ver con el tiempo dedicado al área de autocuidado, concretamente al sueño, habiéndose observado en este tipo de patologías cómo el tiempo de descanso está por debajo de las siete horas recomendadas^19^, fundamentalmente debido al dolor, la fatiga, la limitación de la función articular y muscular y la alteración del estado de ánimo^20^. Nuestros resultados corroboran este aspecto.

Otra área importante que forma parte de un buen equilibrio ocupacional son las actividades laborales. Los participantes de esta investigación perciben también un impacto negativo en el desempeño de aquellas, probablemente, por la presencia de síntomas o por requerir más tiempo para realizar las tareas. Por otro lado, y aunque en la literatura revisada sobre el tema se afirma que la intervención de TO puede reducir la discapacidad laboral y promover el trabajo estable^21^^,^^22^, ninguno de nuestros participantes recibía este tipo de terapia. Estar laboralmente activo aumenta la autoestima, garantiza la independencia financiera, reduce los síntomas de fatiga y mejora la salud mental como resultado de una mayor participación social y menor sentimiento de impotencia^23^.

Los entornos familiar y social resultan determinantes también para la participación en las AVD de las personas con enfermedad reumática, ya que, a través de sus actitudes y acciones, los seres queridos actúan como agentes limitantes o favorecedores en dicha participación^24^. Sin embargo, el 40% de nuestra muestra consideró que la relación con sus familiares y/o amistades había empeorado.

En nuestro conocimiento, este es el primer estudio que analiza el desempeño ocupacional en pacientes con diagnóstico de enfermedad reumática en la población española. El desempeño ocupacional de nuestros participantes, en cuanto a realización y satisfacción, fue bastante bajo. Este peor desempeño podría ser causa (o consecuencia) de la asociación de la enfermedad reumática con una peor capacidad funcional y laboral, y calidad de vida^25^. Futuros estudios podrían aclarar la relación causa-efecto.

Nuestros resultados no arrojan diferencias significativas respecto al equilibrio ocupacional, la participación, el rendimiento y la satisfacción entre participantes según recibieran o no terapia no farmacológica. Por el contrario, un ensayo clínico aleatorizado señala que la TO centrada en los objetivos y prioridades de personas con artritis reumatoide, para mejorar varias limitaciones del desempeño ocupacional (cuidado personal, gestión comunitaria y del hogar, movilidad funcional, recreación pasiva y activa, socialización y trabajo), reduciría la limitación de la actividad y las restricciones de participación, aumentando la calidad de vida de estas personas^26^. Quizás la principal diferencia, respecto a nuestros resultados, haya sido que ningún participante realizó TO. Los sujetos que recibían terapia no farmacológica acudían únicamente a fisioterapia.

Respecto a la participación, se ha descrito ser menor esta en mujeres con artritis reumatoide frente a la población general^27^, ello se explicaría no solo por el dolor y la fatiga causados por la enfermedad, sino también por la preocupación por realizar de forma adecuada las actividades, lo que comprometería su disfrute^27^. Asimismo, en los pacientes con artritis reumatoide son frecuentes los sentimientos de tristeza y vergüenza^17^ lo que, junto al dolor y la fatiga, puede influir negativamente en la realización de aficiones, lo que explicaría que el 81% de nuestros participantes no les dediquen todo el tiempo que les gustaría, con el consiguiente desequilibrio ocupacional en el área de ocio y tiempo libre.

En relación con la edad como factor que pudiera determinar los resultados del desempeño, los datos arrojados por el COMP (respecto a la ejecución) indican peores puntuaciones a mayor edad, pero no respecto a la satisfacción cuyas diferencias no fueron significativas. Este hecho, sin embargo, no fue observado por Wagman y col^9^, que señalan que el desempeño ocupacional es mejor percibido en personas mayores de 65 años.

El presente estudio no está exento de limitaciones. La primera es el reducido tamaño muestral, lo que disminuye la potencia y ha podido afectar a no detectar diferencias en alguna de las variables estudiadas. Una segunda limitación es la procedencia de la muestra, ya que todos los participantes procedían de una única asociación, lo que no permite generalizar los resultados. En tercer lugar, las características clínicas de la muestra solo han permitido incluir cuatro patologías reumáticas, un número escasamente representativo de las más de 200 descritas; serán necesarios futuros estudios que incluyesen mayor número de patologías reumatológicas con un mayor volumen de cada una de ellas para poder comparar las repercusiones sobre el equilibrio ocupacional de cada una de ellas. Aunque se hayan incluido cuatro tipos de patologías reumáticas, el pequeño tamaño de cada una de ellas ha impedido desagregar todos los resultados por patología. Y en cuarto y último lugar, la mayoritaria participación femenina no permite extrapolar los resultados a la población masculina.

Este estudio ha permitido obtener información útil sobre el perfil ocupacional de personas con patología reumática en fases no avanzadas de la enfermedad. Estas personas no presentaron un buen equilibrio ocupacional; este fue percibido como adecuado pero mejorable si encontrasen menos limitaciones al realizar las AVD, ya que no sentían que su desempeño fuera óptimo y no estaban satisfechos con los resultados. A priori, parece que la edad o recibir tratamiento no farmacológico (desde la fisioterapia) no se relacionaron con el nivel de equilibrio ocupacional.

Esta información podría resultar valiosa para diseñar programas de intervención de TO centrada en los intereses y limitaciones de las personas con enfermedad reumática, con el fin de mejorar su equilibrio ocupacional, funcionalidad, desempeño y satisfacción, y así como para disminuir la discapacidad mejorando su participación y calidad de vida.

## References

[1] Phang JK, Kwan YH, Goh H, Tan VIC, Thumboo J, Østbye T (2018). Complementary and alternative medicine for rheumatic diseases: A systematic review of randomized controlled trials. Complement Ther Med.

[2] Sangha O (2000). Epidemiology of rheumatic diseases. Rheumatology (Oxford).

[3] Grupo Cientffico de Ia OMS en Enfermedades Reumaticas (1992). Enfermedades reumaticas: informe de un grupo cientifico de Ia OMS.

[4] Carmona L, Loza E (2015). Epidemiología. Dossier de prensa de la Sociedad Española de Reumatología.

[5] Steultjens EMJ, Dekker J, Bouter LM, van Schaardenburg D, van Kuyk MAH, van den Ende CHM (2004). Occupational therapy for rheumatoid arthritis. Cochrane Database Syst Rev.

[6] Roll SC, Hardison ME (2017). Effectiveness of occupational therapy interventions for adults with musculo-skeletal conditions of the forearm, wrist, and hand: A systematic review. Am J Occup Ther.

[7] American Occupational Therapy Association (AOTA) (2008). Marco de trabajo para la práctica de terapia ocupacional. Dominio y proceso. Adaptación al español del documento: American Occupational Therapy Asociation. Occupational therapy practice framework: Domain and process. Am J Occup Ther.

[8] Stamm T, Wright J, Machold K, Sadlo G, Smolen J (2004). Occupational balance of women with rheumatoid arthritis: A qualitative study. Musculoskeletal Care.

[9] Wagman P, Ahlstrand I, Björk M, Håkansson C (2020). Occupational balance and its association with life satisfaction in men and women with rheumatoid arthritis. Musculoskeletal Care.

[10] Ekelman BA, Hooker L, Davis A, Klan J, Newburn D, Detwiler K (2014). Occupational therapy interventions for adults with rheumatoid arthritis: An appraisal of the evidence. Occup Ther Health Care.

[11] Peral Gómez P, López S, Pastor MA, Abad E, Valera D, Hákansson C (2021). Cultural adaptation and psychometric properties of the spanish version of the occupational balance questionnaire: an instrument for occupation-based research. Int J Environ Res Public Health.

[12] Post MWM, Witte LP, Reichrath E, Verdonschot MM, Wijlhuizen GJ, Perenboom RJM (2008). Development and validation of impact-s, an ICF-based questionnaire to measure activities and participation. J Rehabil Med.

[13] Eyssen ICJM, Steultjens MPM, Oud TAM, Bol EM, Maasdam A, Dekker J (2011). Responsiveness of the Canadian Occupational Performance Measure. J Rehabil Res Dev.

[14] Sarabia CM, Alconero AR (2019). Claves para el diseño y validación de cuestionarios en Ciencias de la Salud. Enferm Cardiol.

[15] Gray BH, Cooke RA, Tannenbaum AS (1978). Research involving human subjects. Science.

[16] Wagman P, Håkansson C, Björklund A (2012). Occupational balance as used in occupational therapy: A concept analysis. Scand J Occup Ther.

[17] Katz PP, Morris A, Yelin EH (2006). Prevalence and predictors of disability in valued life activities among individuals with rheumatoid arthritis. Ann Rheum Dis.

[18] Ahlstrand I, Björk M, Thyberg I, Börsbo B, Falkmer T (2012). Pain and daily activities in rheumatoid arthritis. Disabil Rehabil.

[19] Feehan LM, Lu N, Xie H, Li LC (2020). Twenty-four hour-activity and sleep profiles for adults living with arthritis: Habits matter. Arthritis Care Res Hoboken.

[20] Ma KS, Maria M, Ralda I, Veeravalli JJ, Wang L, Thota E (2022). Patients with juvenile idiopathic arthritis are at increased risk for obstructive sleep apnoea: A population-based cohort study. Eur J Orthod.

[21] Macedo AM, Oakley SP, Panayi GS, Kirkham BW (2009). Functional and work outcomes improve in patients with rheumatoid arthritis who receive targeted, comprehensive occupational therapy. Arthritis Care Res.

[22] Madsen CMT, Christensen JR, Bremander A, Primdahl J (2023). Perceived challenges at work and need for professional support among people with inflammatory arthritis - a qualitative interview study. Scand J Occup Ther.

[B23] Papakonstantinou D (2021). Work disability and rheumatoid arthritis: Predictive factors. Work.

[B24] Bergström M, Sverker A, Larsson Ranada Å, Valtersson E, Thyberg I, Östlund G (2020). Significant others' influence on participation in everyday life- the perspectives of persons with early diagnosed rheumatoid arthritis. Disabil Rehabil.

[25] Nikiphorou E, Ramiro S, Heijde D, Norton S, Moltó A, Dougados M (2018). Association of comorbidities in spondyloarthritis with poor function, work disability, and quality of life: results from the assessment of SpondyloArthritis International Society Comorbidities in Spondyloarthritis study. Arthritis Care Res.

[26] Tonga E, Düger T, Karatas M (2016). Effectiveness of client-centered occupational therapy in patients with rheumatoid arthritis: Exploratory randomized controlled trial. Arch Rheumatol.

[27] Reinseth L, Kjeken I, Uhlig T, Espnes G (2012). Participation in committed and discretionary activities and quality of life in women with rheumatoid arthritis. Br J Occup Ther.

